# Into the well—A close look at the complex structures of a microtiter biofilm and the crystal violet assay

**DOI:** 10.1016/j.bioflm.2019.100006

**Published:** 2019-09-12

**Authors:** Kasper Nørskov Kragh, Maria Alhede, Lasse Kvich, Thomas Bjarnsholt

**Affiliations:** a Costerton Biofilm Center, Department of Immunology and Microbiology, Faculty of Health Sciences University of Copenhagen, Blegdamsvej 3B, 2200, Copenhagen, Denmark; b Department of Clinical Microbiology, Henrik Harpestrengs Vej 4A, Rigshospitalet, 2100, Copenhagen, Denmark

**Keywords:** Biofilm, *Pseudomonas aeruginosa*, Microtiter assay, Crystal violet, Confocal laser scanning microscopy, *In vitro* validation

## Abstract

The microtiter assay is one of the most widely used methods for assessing biofilm formation. Though it has high throughput, this assay is known for its substantial deviation from experiment to experiment, and even from well to well. Since the assay constitutes one of the pillars of biofilm research, it was decided to examine the wells of a microtiter plate directly during growth, treatment, and the steps involved in crystal violet (CV) measurements.

An inverted Zeiss LSM 880 confocal laser scanning microscope was used to visualize and quantify biomass directly in the wells of the microtiter plate. Green fluorescent protein-tagged *Pseudomonas aeruginosa,* PAO1, and live/dead stains were used to assess the structure, state, and position of biomass build-up. Microscopic observations were compared with colony-forming unit (CFU) and CV measurements.

The development and the structured architecture of biomass was observed in real-time in the wells. Three-dimensional images of biomass were obtained from all of the microtiter wells; these showed variations from well to well. CV staining showed large variations in remaining biomass, depending on the method selected to remove the supernatant prior to CV staining (i.e. pipetting or manually discarding the fluid by inversion, washed or unwashed wells). Colony-forming unit counts or live/dead staining used to evaluate biomass with or without antibiotic treatment proved imprecise due to aggregation, limited removal of biomass, and overestimation of dead staining.

The highly structured microenvironment of biomass in microtiter wells needs to be considered when designing and analyzing experiments. When using microtiter plates, stochastic variation due to growth and handling may lead to flawed conclusions. It is therefore recommended that this assay be used as a screening tool rather than as a stand-alone experimental tool.

## Introduction

In microtiter biofilm assays, biofilms are grown on surfaces of polystyrene wells filled with static media. These assays were originally introduced by O’Toole and Kolter [1]. This widely used model constitutes a high throughput system, which is suitable for screening the biofilm-forming ability of bacteria as well as for testing *anti*-biofilm compounds [2]. The system uses flat-bottomed or U-shaped wells, which are able to hold approximately 400 μL in the case of 96-well plates. When these wells are filled with 150–300 μL of media and inoculated with small amounts of bacteria, biofilm will form within the first 24 h on the sides and bottom of the well as well as at the air–liquid interface [3]. The assay can be used with all kinds of media, from well-defined (e.g. 0.5% w/v glucose) minimal media to richer, more complex media such as lysogeny broth (LB) or tryptic soy broth [[3], [4], [5]]. During or after the growth of biofilm, the media can be supplemented with different kinds of antimicrobial or *anti*-biofilm agents. Biomass build-up in the wells is commonly quantified by means of crystal violet (CV) staining, CFUs to assess the killing effectiveness, or propidium iodide (PI) staining with the intent to evaluate the amount of dead bacteria [4,6,7].

Though, high throughput, this assay is known for its great variation from experiment to experiment, and even from well to well. This lack of reproducibility is often attributed to differences in handling by laboratory personnel, differences in the inoculum, or variations in growth in the individual wells [8]. Differences in adhesion between different batches of PVC microtiter plates have also been reported, which could also account for the poor reproducibility [9,10]. Although the 96-well microtiter assay has been further developed in the form of the Calgary system, is the former is still predominant in biofilm research [7,11,12]. The Calgary system, also referred to the MBEC model, uses the same type of 96-well microtiter plates, but has a lid with 96 pegs that protrude down into the media. Cells may adhere to the pegs, as well as to the sides of the wells, and proliferate into surface-attached biofilm. The peg lid can be transferred to new plates with fresh media, be exposed to treatment, or be rinsed to quantify biomass.

In other biofilm models, such as the continuous-culture flow-cell system [13], large variations in biofilm biomass, structure, and composition have been reported due to small variations in microenvironmental conditions or small procedural variations [14,15]. Such variations in the microenvironment include oxygen gradients or variations in the carbon sources [[16], [17], [18]]. The composition of the initial inoculum has also been shown to have an impact on the development of the biofilm structure [14]. Due to the static conditions of the media in the microtiter wells, steep oxygen gradients are likely to develop from the surface of the media down through the well during an experiment. This system of static media may also result in a large planktonic population in the bulk fluid of the well, which will consume their part of the available carbon and oxygen, competing with the attached biofilm bacteria.

Although microtiter biofilm assays using 96-well plates are widely used, there are limited knowledge regarding the physiology, mechanisms, and dynamics within the well itself when biofilm is growing. Where and how is biomass positioned? Is the growth homogeneously spread over the surface of the well? What is the origin and state of biomass stained by CV in the wells? In addition, what contributes to the variations in results of this assay?

We aimed to answer these questions through direct high-resolution confocal 3D imaging and measuring biomass within wells at various stages of growth and procedures during biofilm formation, coupled with standardized methods such as CFU and CV quantification.

## Results

### Growth in wells for 24–72 h

**Fig. 1:**
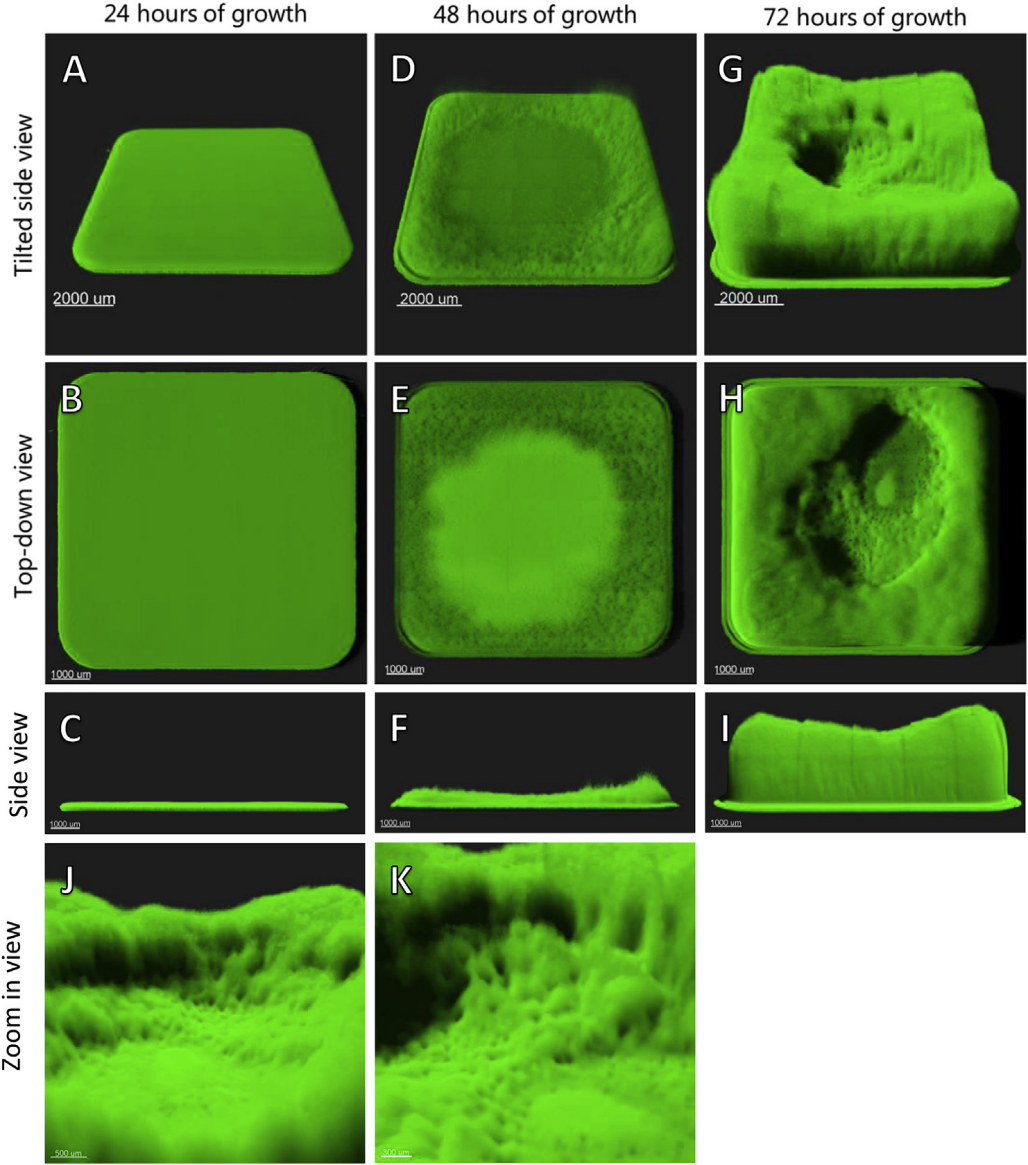
Representative 3D projections of undisturbed *P. aeruginosa* biomass at the bottom of microtiter wells grown for 24, 48, and 72 h. Images consist of 7 × 7 stitched z-stack images with 100x magnification. A–C) A 24-h old biomass. D–F) A 48-h old biomass. G–I) A 72-h old biomass. J-K) Zoom in on structures present in 72 h old biomass.

**Fig. 2:**
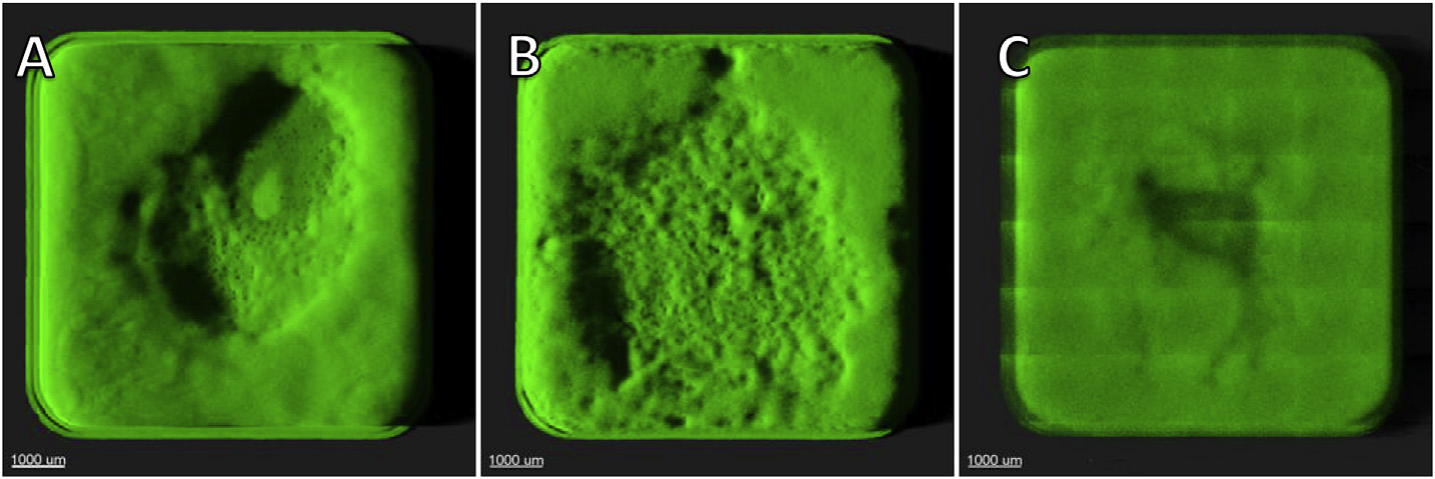
A-C) 3D projections of three neighboring wells each containing 72-h old biofilm. All three wells were inoculated, grown, and imaged in the same manner.

Undisturbed biomass grown in LB media in 96-well microtiter plates was observed every 24 h using confocal laser scanning microscopy (CLSM) to evaluate the structure and amount of biomass. After 24 h of growth, a 50–100 μm thick flat lawn of biomass with minimal structure could be observed (Fig. 1A–C). After 48 h, the biomass in the wells appeared more structured, with biomass rising towards the edges of the wells and a flattened surface in the central part of the well (Fig. 1D–F). After 72 h of growth, the wells exhibited a heterogeneous structured biomass with cracks, ravines, and towering biomass toward the edges of the well (Fig. 1G–K). In wells in which biomass had been growing for 24 h, it appeared consistent across wells, whereas more heterogeneity was observed across wells after 48 h of growth. The biomass structure in wells in which it had been growing for 72 h was highly diversified across wells (Fig. 2).

**Fig. 3:**
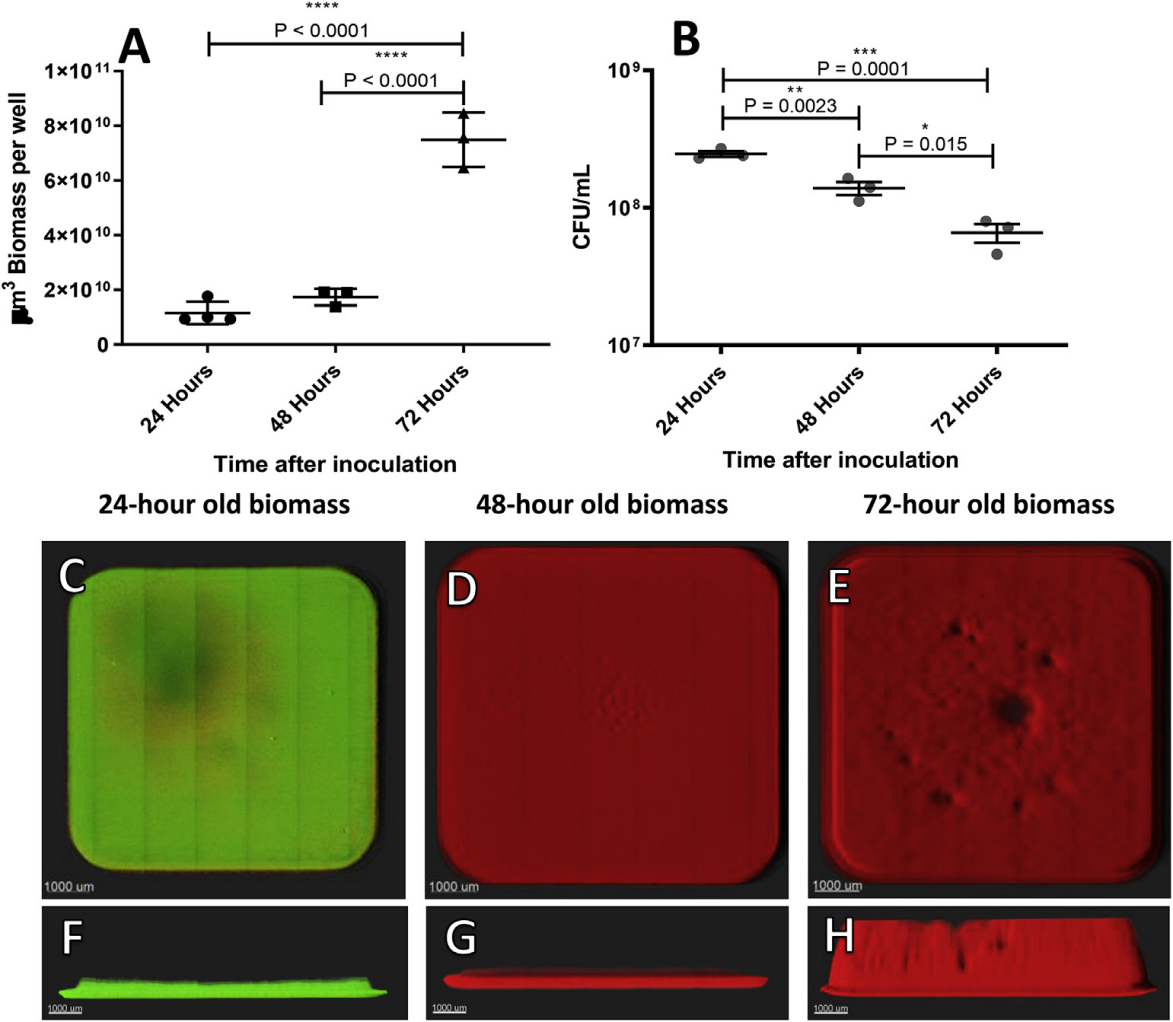
A) Quantitative measurements of undisturbed *P. aeruginosa* biomass in 24-, 48-, and 72-h old wells. There was significantly more biomass (μm [3]) in 72-h old wells than in 24- and 48-h old wells. B) CFU count from undisturbed wells at the three timepoints. C–E) 3D projections of undisturbed *P. aeruginosa* biomass stained with live/dead stain. F–H) Side view of *P. aeruginosa* biomass stained with live/dead stain.

Based on CLSM images, biomass was quantified in wells at all three points in time. No significant change in the amount of biomass between 24 and 48 h was observed. Biomass grown for 72 h was significantly larger than the 24- and 48-h old biomass (P < 0.0001 and P < 0.0001, respectively) (Fig. 3A). The quantified biomass exhibited an increasing deviation in biomass at 72 h (SD ± 9.97 × 109 μm^3^) as compared to the biomass at 24 (SD ± 4.11 × 109 μm^3^) and 48 h (SD ± 3.06 × 109 μm^3^). The deviation in height increased from 24 h (SD ± 124 μm) to 48 h (SD ± 408 μm) and further after 72 h (SD ± 1107 μm).

**Fig. 4:**
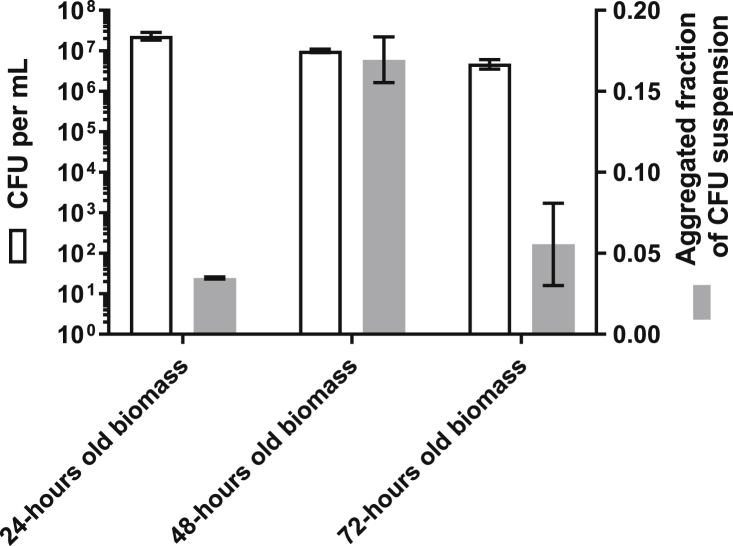
White columns represent CFU counts from removed biomass in microtiter wells grown for 24, 48, or 72 h. The wells were scraped and ultra-sonicated to remove the biomass. The removed biomass was then degassed and ultra-sonicated before serial dilution was conducted prior to CFU determination. Prior to serial dilution, each sample was evaluated for the amount of biomass bound in aggregates larger than 100 μm^3^. The fraction of the total biomass bound in aggregates is represented in the gray columns.

To assess how much of the biomass contained viable cells, the total number of CFUs were counted in undisturbed wells after 24, 48, and 72 h of growth. There was a decline in CFUs per mL from a 24-h old biomass to a 48-h old biomass (P = 0.0023). Decreased recovery from 48 to 72 h of growth was likewise observed (P = 0.015) (Fig. 3B). Biomass removed from the wells was analyzed for aggregation following degassing and sonication to assess how well the CFUs reflected a definite single cell count. The fraction of the biomass bound in aggregates (>100 μm [3]) ranged from 0.045 to 0.165 of the total 24 and 48-h old biomass, respectively, when removed from the wells (Fig. 4). Live/dead stain was applied to the wells to ascertain the viability of biomass grown for 24–72 h. The 24-h old biomass exhibited a Syto9-stained (viable) lawn with small patches of PI-stained (non-viable) cells (Fig. 3C). After 48 and 72 h of growth, the PI-stained biomass was much more pronounced than at 24 h (Fig. 2D and E).

### Removal of cells from microtiter wells

Several common methods for removing biomass from microtiter wells prior to CFU count were evaluated using CLSM imaging and biomass quantification.

**Fig. 5:**
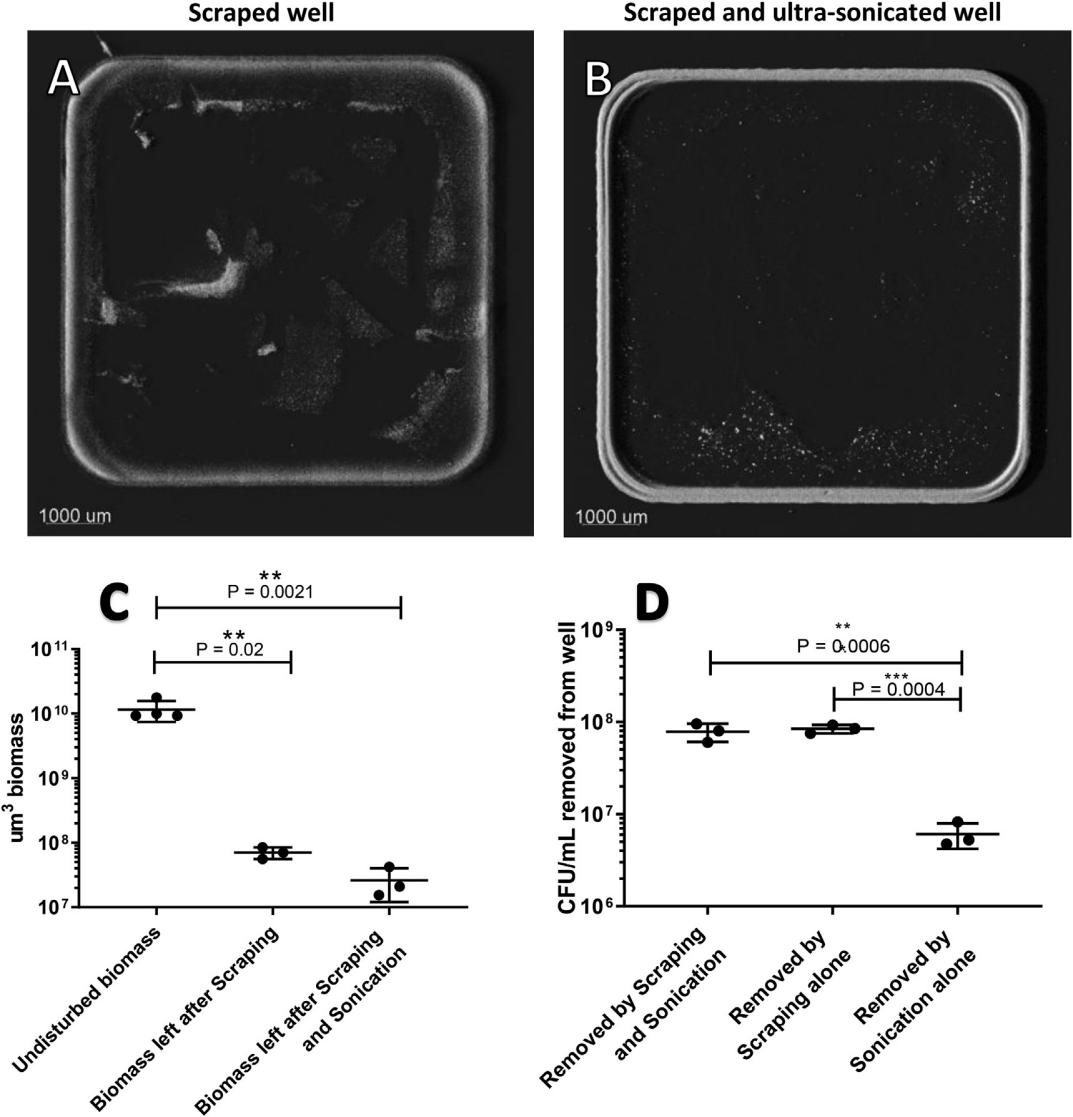
Removal of total biomass for the CFU count by means of scraping or both scraping and ultra-sonication. A) 3D projections of remaining biomass after thorough scraping. B) 3D projections of remaining biomass after thorough scraping followed by ultra-sonication. Images consist of 7 × 7 stitched z-stack images with a 100x magnification. C) Quantitative measurements of biomass left in the wells after scraping or both scraping and ultra-sonication, compared to the undisturbed control. D) The number of CFUs removed by scraping alone, ultra-sonication alone, or by the two methods combined.

Remaining biomass from the wells was imaged and quantified after scraping or both scraping and ultra-sonication. There was visible biomass left at the edges and in the central parts of the wells when biomass was removed by scraping (Fig. 5A). When ultra-sonication was used to remove biomass from the surface, there was still visible biomass at the edges of the wells (Fig. 5B).

Scraping or scraping and ultra-sonication removed a significant amount of biomass compared to wells which were not scraped or subjected to ultra-sonication (P = 0.002 and P = 0.021). The addition of ultra-sonication as a step to remove biomass did not improve the removal of biomass compared to wells that had only been scraped (P = 0.999) (Fig. 5C).

Colony-forming units were used to estimate how many viable cells where removed from the wells by scraping, ultra-sonication, or scraping and ultra-sonication. Scraping and ultra-sonication removed the same number of cells as scraping alone (P = 0.81). Ultra-sonication alone removed fewer than scraping or scraping and ultra-sonication (P = 0.0004 and P = 0.0006, respectively) (Fig. 5D).

### The effect of common steps of crystal violet assay on biofilm in 24-h old biomass

Wells were imaged with CLSM and biomass was quantified at different steps in the CV procedure to investigate how it affected the biofilm at the bottom of wells. Parallel wells were stained with and without CV. The unstained well was used to compare the number of CFUs left in the attached biofilm.

**Fig. 6:**
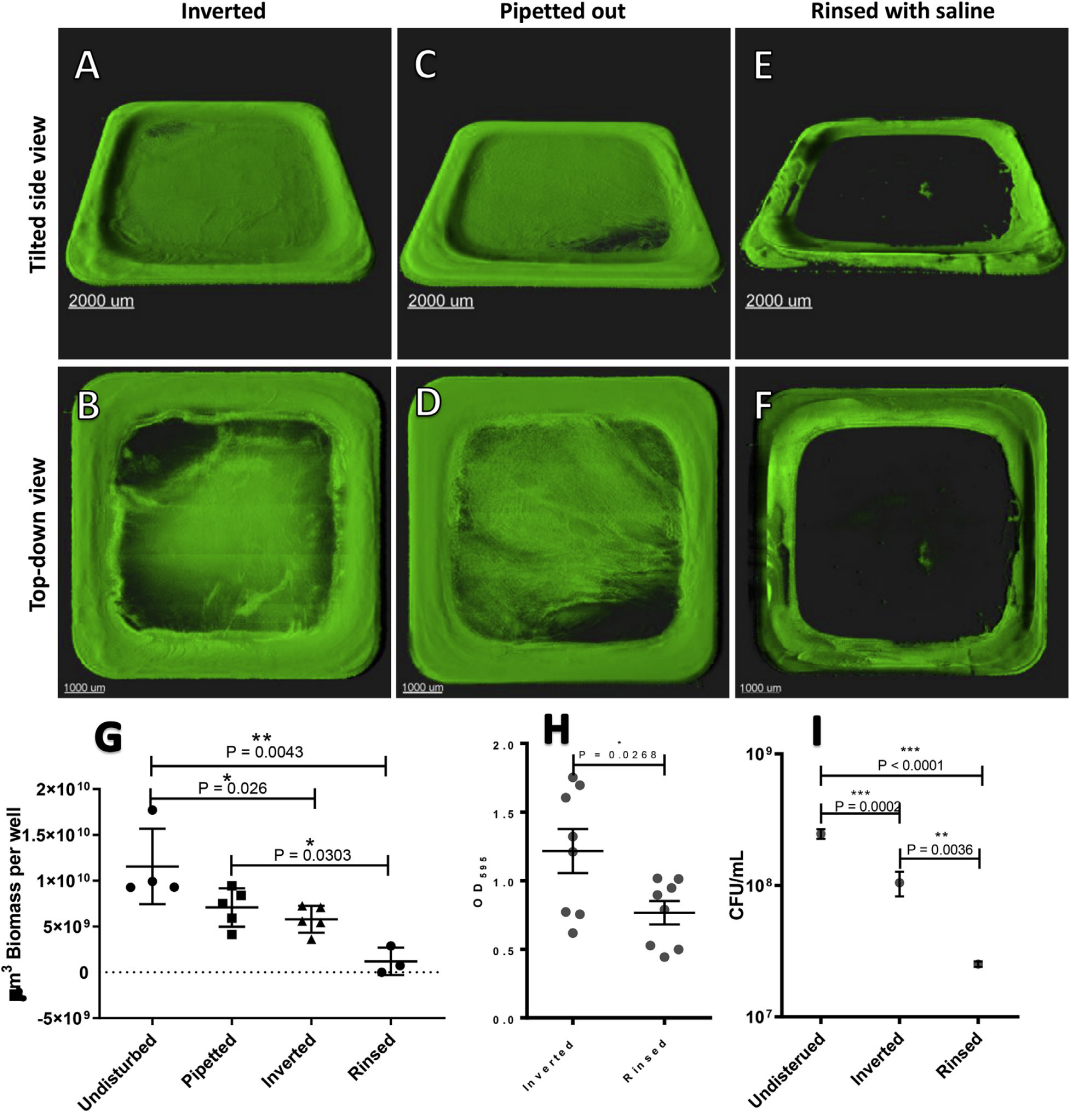
Representative 3D projections of 24-h old biomass remaining at the bottom of microtiter wells after the supernatant had been removed as part of the crystal violet staining procedure, by means of inversion, pipetting, or pipetting followed by a rinse with saline. Images consist of 7 × 7 stitched z-stack images with a 100x magnification. A–B) Biomass remaining after the removal of the supernatant by means of inversion. C–D) Biomass remaining after removal of the supernatant by means of pipetting. E–F) Biomass remaining after rinsing with saline. G) Quantitative measurements of 24-h old biomass either undisturbed or remaining at the bottom of microtiter wells after the supernatant had been removed as part of the crystal violet staining procedure, by means of inversion, pipetting, or pipetting followed by a rinse with saline. H) OD_595_ measurement of crystal violet staining of remaining biomass after the supernatant was removed either by inversion or pipetting followed by a rinse with saline. I) CFU count for the biomass in undisturbed wells or remaining in the wells after inversion or pipetting and rinse. (For interpretation of the references to color in this figure legend, the reader is referred to the Web version of this article.)

If the supernatant was removed by inversion of the plate, a thin layer of biomass was observed in the central part of the wells along with biomass towards the edges (Fig. 6A and B). Compared to the undisrupted biomass grown for in 24 h (Fig. 1A and B), the inverted wells had lost the flat lawn in the central part of the well. In wells where the supernatant was removed by careful pipetting, it was noted that the biomass residue showed signs of the removal of supernatant created by the tip of the pipette (Fig. 6C and D). The structure of the biomass left by the pipette was otherwise comparable with the wells that were emptied by inversion, with a flat central part and more biomass remaining towards the edges. In wells that were inverted and subsequently rinsed twice with saline, only a band along the very edges of the wells was observed, with small patches of biomass scattered in the central part of the wells (Fig. 6E and F).

Biomass was quantified based on CLSM images of wells where the supernatant was removed either by inversion or pipetting, with or without rinsing. For the purpose of comparison, the biomass in the undisturbed wells was included in Fig. 6G. There was no significant difference in the amount of biomass in undisturbed wells and pipetted wells. There was significantly less biomass in wells washed with saline and in wells that had been inverted compared to the undisturbed wells (P = 0.0043 and P = 0.026, respectively). Wells that had been emptied by pipetting contained significantly more biomass than those that had been washed with saline (P = 0.0303) (Fig. 6G).

Wells were stained with CV after the supernatant had been removed, either by inversion or pipetting, followed by rinsing with saline. Crystal violet quantification showed a significantly larger amount of biomass left in wells that had been inverted than in those that had been pipetted and rinsed (P = 0.0268) (Fig. 6H).

Colony-forming units were used to evaluate the viable part of the attached biofilm after the supernatant had been removed by inversion or pipetted and rinsed and were compared with undisturbed wells. There was significant fewer CFUs in wells that were pipetted and rinsed than in wells that had been inverted (P = 0.0036) (Fig. 6I). A significant number of cells were removed from the undisturbed wells by both inversion and pipetting and rinse (P = 0.0002 and P < 0.0001, respectively) (Fig. 6I).

### Tobramycin treatment of 24–72-h old biofilm

To investigate how antibiotic treatment effects the structure of biomass in microtiter plates, 24-h old biomass was treated with 10 μg mL^−1^ tobramycin and stained with Syto9 and PI to indicate live and dead cells while being imaged using CLSM.

**Fig. 7:**
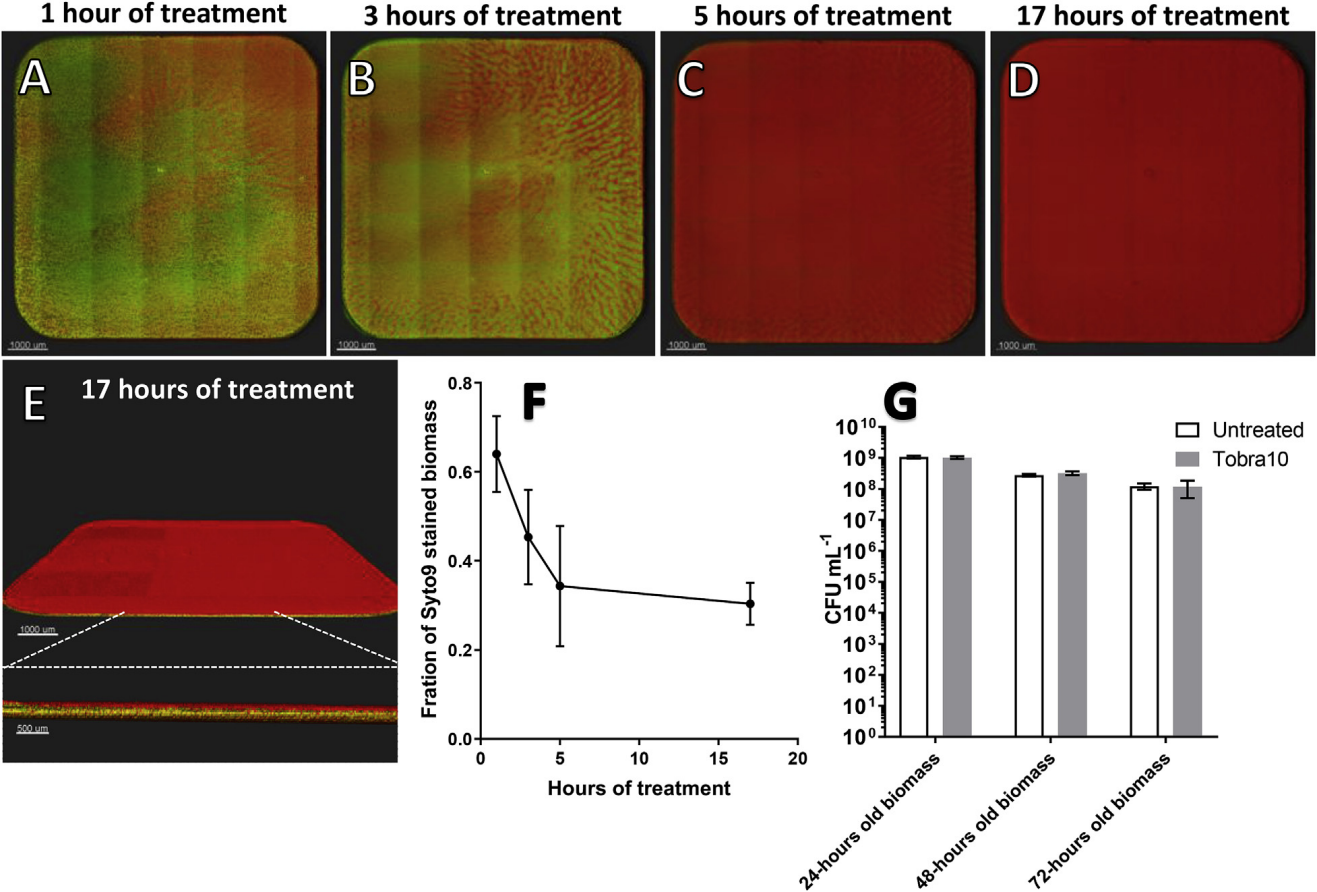
3D projections of 24-h old biomass treated with 10 μg mL^−1^ tobramycin and stained with live/dead stains. A) The biofilm 1 h after the beginning of treatment. B) The biofilm 3 h after the beginning of treatment. C) The biofilm 5 h after the beginning of treatment. D) The biofilm 17 h after the beginning of treatment. E) Side view of 17-h old treated biofilm, also shown in 5D. Zoom showing the top layer of PI-stained biomass on top of Syto9-stained biomass. F) Fraction of biomass stained green with Syto9. Biomass of both PI- and Syto9-stained cells were quantified, and the part stained with Syto9 was taken as a fraction of the total. G) CFU after 17 h of Tobramycin (10 μg mL^−1^) treatment or untreated biomass in microtiter wells grown 24, 48, or 72 h before treatment. (For interpretation of the references to color in this figure legend, the reader is referred to the Web version of this article.)

After 1 h of treatment with 10 μg mL^−1^ of tobramycin, several areas of the biomass exhibited slightly reddish coloration (Fig. 7A). The PI staining had intensified after 3 h of treatment, with structured red lines throughout the wells (Fig. 7B). After 5 h of treatment, the biomass was predominantly stained red by PI, with a few green patches along the edges (Fig. 7C). After 17 h of treatment, the biomass was strongly stained with PI (Fig. 7D). The PI predominantly stained the upper layers of biomass after 17 h of treatment, as may be observed from the side views in Fig. 7E. The total amount of biomass, as well as its structure, seem unaffected by the treatment.

The fraction of 24-h old biomass stained with Syto9 (live cells) exhibited a steep decline during the first 1–5 h of treatment and more restricted decline after 5 and 17 h of treatment (Fig. 7F). The treatment with 10 μg mL^−1^ of tobramycin did not kill a significant number of the attached biofilm compared to untreated biofilm when assessed by means of CFU counts (Fig. 7G). The uncertainties this involves are described above.

## Discussion

Our initial observation of biomass grown in a 96-well microtiter plate for 24 h, was of a flat, uniform biofilm at the bottom of the well with relatively little growth at the air-liquid interface, which corresponds with what is commonly described [1,19]. Over time, we observed how biofilms developed into a much more structured biofilm with complex shapes. The structuring of the biofilm over time is in accordance with other *in vitro* biofilm models, such as the continuous-culture flow-cell system [20]. In these systems, structured biofilm growth has been attributed to the competition for nutrients in the media as height and structure can provide a growth advantage by elevating the biofilm from the dense lawn [16,21,22]. Although the microenvironment in a microtiter well does not have a flow of media, nutrition and oxygen gradients will occur from the air interface towards the bottom, resulting in the same growth advantage over time.

Our general observation was that the heterogeneity of the biomass in the wells increased dramatically over time as each developed seemingly stochastically differently, depending on the microenvironment in the individual well. This heterogeneous growth may contribute to the deviations often found in this model as the local microenvironment in each well may influence growth, strength, and resilience [[23], [24], [25]].

The biomass at the bottom of the wells continues to grow in biomass over 72 h; however, CFUs recovered from this growing biomass decreased in number. The number of countable cells was reduced by approximately 50% from 24 to 48 h and by a further 50% from 48 to 72 h. This may have been a result of a decreasing number of CFUs, though it may also have been a result of increased bacterial aggregation and the subsequent inability to break these aggregates apart prior to CFU determination. It is in most cases impossible to dissolve bacterial aggregates into a homogeneous solution of single cells [26]. This means that a bacterial aggregate will only show as one CFU when serial dilutions are used, thereby leading to an underestimation of the presence of viable cells. By analyzing the amount of aggregated biomass in the biomass removed after sonication, a fraction up to 0.165 of the total biomass could be found as aggregates above 100 μm [3] in size. Thus, the decline in CFUs recovered from the wells was not necessarily an indication of bacteria dying over time but may rather be due to procedural limitations. Colony-forming unit counts from microtiter wells have additional limitations due to the process involved in removing biomass. In our study, we observed how difficult it is to remove the entire biomass from the well for the purpose of undertaking CFU counts. Previously, scraping and ultra-sonication have been recommended for removing attached biomass from microtiter wells. In this study, scraping removed approximately 90% of attached biomass from the bottom of the wells, and ultra-sonication did not remove significantly more cells. Combining these methods left approximately 5% of the biomass in the wells, mostly at the very edge. Scraping introduced mechanical interaction with the biofilm while handling by laboratory personnel introduced variation, as seen in the representative image of a thoroughly scraped well in Fig. 5A. This can lead to a misinterpretation if it is expected that all viable cells have been removed. We were surprised to find that ultra-sonication did not have any significant added effect over and above scraping, though the limitations of ultra-sonication in completely removing biofilm have previously been described [27,28]. This observation of course also introduces uncertainties regarding our own CFU counts as presented in this study, and should therefore only be used as a comparative indicator within the same experiment.

We were able to observe how even experienced laboratory personnel produced varying results due to stochastic variations during the removal of the supernatant and of the loosely bound biomass. It was clear that both pipetting and inversion of the supernatant removed biomass heterogeneously and left signs of the removal, especially in the case of pipetting. By rinsing the wells with saline, a large amount of biomass was removed, but this did not remove the variation between wells completely. Crystal violet staining of the wells produced optical density (OD) measurements with a standard deviation corresponding to ~30%. We observed how biomass that had incubated for 72 h had the same or, in some instances, less biomass bound to the surface of the wells than did 24-h old biomass. This was even though the undisturbed 72-h old biomass contained approximately eight times more biomass than the 24-h old biomass, which seemed to be bound loosely and could easily be removed. This fits with previous observations of a large part of the biomass being precipitated [7,11]. Generally, the proportion of biomass bound and remaining in the wells after removal of the supernatant and the rinse seemed to be quite stochastic, which may explain the variation often observed in this system. The alternative approach to quantifying biofilm formation using the Calgary peg-lid system may be a viable substitute in order to minimize this stochastic deviance that results from loosely bound biomass and variable supernatant removal [7]. The Calgary system uses pegs on which the primarily attached biomass is found, and precipitate is removed as a result of gravity [7].

To investigate the effects of antibiotic treatment on biofilm in the microtiter plate assay and how the effectiveness of the treatment was influenced by the structure of the biofilm in the wells, we applied the aminoglycoside, tobramycin. Tobramycin targets the ribosomal subunit, and therefore has a limited effect on slow-growing biofilm cells [29,30]. We used a concentration equal to 10 times the minimal inhibitory concentration (MIC) for *P. aeruginosa.* [31] One hour following treatment, PI-stained patches of bacteria appeared, which intensified and spread in a clear striped pattern after 3 h of treatment. This pattern may be the result of smaller local variations in growth or in the microenvironment and may be due to structural heterogeneity. However, this phenomenon requires further investigation. Five hours following treatment, the surface of the bottom-lying biomass was predominantly stained with PI, especially the top layers, which accounted for approximately 61% for the biomass stained with PI. This PI staining of the top layer as a result of tobramycin treatment of biofilm has previously been described in flow cells [32]. After 17 h of treatment, the biomass appeared more densely stained with PI, and this portion of the biomass accounted for approximately 66% of the total. At the magnification used for the well imaging as a whole, it is difficult to distinguish single cells from extracellular stained DNA. A high incidence of extracellular PI staining has been reported, which may explain the overestimation of PI-stained pixels during the quantification procedure [33].

A CFU count from the wells, with all its limitations, as previously described, showed that there was no significant killing of cells by the tobramycin compared to untreated wells. This were the case for the 24-, 48- and 72-h old biofilms. This is in accordance with the acknowledged understanding of biofilm’s tolerance to antibiotics in microtiter plate wells [34]. Thus, the observation of PI staining on the surface may merely account for a superficial killing of a limited part of the whole population or may be an overestimation of the killing, as discussed above. This highlights the limited quantitative value of using of live/dead staining for microtiter plate assays [35].

In this study, we thoroughly investigated the 96-well microtiter-plate biofilm assay by direct observation using CLSM in combination with techniques commonly used in biofilm investigations. Our findings suggest that even with this simple biofilm model, complex structures emerge over time as the biofilm matures and the microenvironment becomes more stratified. The biofilm at the bottom of the well may consist of a mixture of tightly attached biomass and loosely bound biomass with a more undefined origin. When emptying the wells of supernatant, this mixture of loose and bound, structured and unstructured biomass can result in highly fluctuating amounts of remaining biomass for quantification. This, in combination with the human factor during the removal of the supernatant, can explain the large variations often reported in microtiter experiments.

The microtiter biofilm assay involves several pitfalls and can result in scattered results due to heterogeneous structured growth, disruptive procedures, and shortcomings in quantifying biomass and viable cells. That said, the microtiter biofilm assay is a fast, high-throughput screening tool with great value for microbiologists, though it is important to appreciate the complexity of even the simplest biofilm model and its limitations in simulating real-world biofilms. It is our hope that our findings will act as an eye-opener to the complexity of this rather simple biofilm model, as well as stress the limitations and pitfalls of this model.

## Method and materials

### Strains and growth conditions

All experiments were conducted using *P. aeruginosa*, PAO1, wild-type strain obtained from the *Pseudomonas* Genetic Stock Center (strain PAO0001 http://www.pseudomonas.med.ecu.edu). For visualization a green fluorescent protein-tagged version was used. This strain had previously been tagged with pMPR9^36^. For visualization of viability, the untagged wild-type strain was stained with live/dead staining consisting of the stains Syto9 (Life Technologies, USA) and PI (Sigma-Aldrich, USA). All experiments were performed at 37 °C in LB media (BD, USA).

Microtiter plates were inoculated from LB overnight cultures started according to Kragh et al. [8] The overnight cultures was diluted to an OD_600_ of 0.005 in LB followed by the transfer of 200 μl to each well investigated in 96-well flat-bottomed Ibidi μ-Plate (Ibidi, Germany). These multi well plates were used as their bottoms are made of a 180 μm thin polymer with a refractive index identical to a #1.5 glass coverslip [37]. These particular plates have semi-squared wells with softly rounded corners, but are otherwise completely comparable with common circular shaped wells. Plates was inoculated for at least 24 h unless otherwise stated.

### Microscopy

For structural analysis, plates where incubated for between 24 and 72 h before imaging was carried out. Whole wells were imaged using an inverted Zeiss LSM 880 running Zen 2.1 (Zeiss, Germany). Wells were imaged using a 488 nm laser for excitation, a 495–550 nm emission filter for GFP and Syto9, and a 561 nm laser and a 595–650 nm emission filter for Pl. Images were taken using a 7 × 7 tilescan approach with an overlap of 20% between tiles. The final dimensions of the images were 9068 μm (X), 9068 μm (Y), and 465 μm (Z). Images were processed in Imaris 9.2 (Bitplane, Switzerland) for both qualitative 3D projections and for biomass quantification using the Measurement Pro addon in Imaris. Imaris utilizes a pixel quantitative approach to measure biomass.

### Colony-forming unit count

**Fig. 8:**
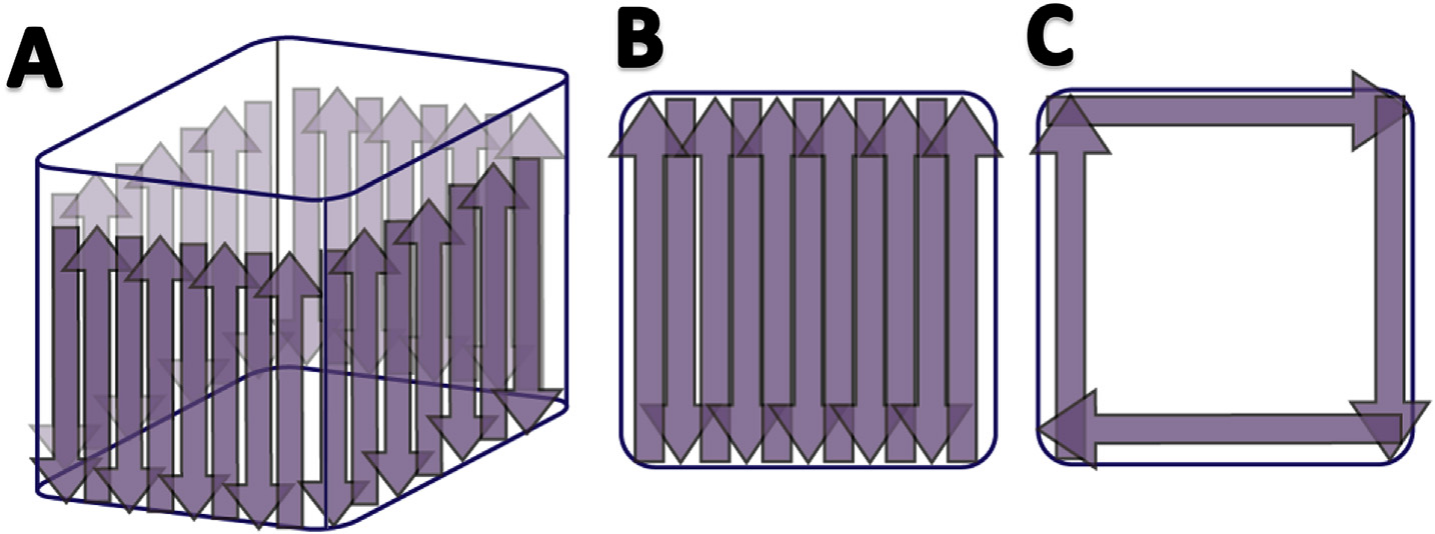
Schematic drawing of scraping procedure. A) Firstly, all surfaces of the walls were scraped in an up-and-down motion while pumping with the pipet. B) Secondly, the bottom were scraped in a back-and-forth motion while pumping with the pipet. C) Finally, the edge between the walls and bottom and the corners, were scraped while pumping the pipet, before the supernatant was removed, followed by another suction to insure removal of all fluid., followed by ultra-sonication (230 VAC, Branson, USA) of the whole plate in a regime of 5 min degassing followed by 5 min of ultra-sonication. The degassing step was used to remove excess gas build-up and bubbles in the samples, which could otherwise inhibits the effect of the ultra-sonication treatment. Biomass was subsequently pipetted out, before it was 10-fold serial diluted and plated on LB plates (1.5% agar). The effectiveness of biomass removal by means of both scraping and ultra-sonication was evaluated using CLSM, as described above.

Colony-forming units were quantified on the basis of biomass removed from wells. Wells were thoroughly and systematically scraped around the walls, bottom and at the edge as pictured in Fig. 8.

The degree of aggregation in the supernatant following degassing and ultra-sonication was assed as described by Kragh et al., 2017 [26]. With the use of CLSM, a 10x dilution of the supernatant was imaged in a μ-slide IV Flat (Ibidi, Germany). Through a quantitative pixel 3 analysis in Imaris, as described above, combined with a size differentiation protocol, aggregated biomass with a volume >100 μm^3^ can be separated from single cells and small clustered biomass <100 μm^3^. The fraction between the two populations can give an indication of the degree of aggregation in the sample.

### Crystal violet staining

Attached biomass was stained by removing the supernatant, either by pipetting or inverting the plates in a turnout motion. Wells were either stained directly or rinsed with saline to remove residual unattached biomass prior to staining. To stain the wells, 300 μl 0.1% v/v CV for 15 min was used. The CV was removed by inverting the plate onto paper towels followed by a rinse with saline to remove residuel CV. Bound CV in the biomass was extracted by filling the wells with 300 μl 96.5% v/v EtOH. The OD of released CV was quantified in a Victor ×4 plate reader (PerkinElmer, USA) at 595 nm in a 0.1 s scan. Parallel to CV staining, biomass was quantified during all steps using CLSM, as described above.

### Tobramycin treatment

Twenty-four hour old wells were treated with 10 μg mL^−1^ tobramycin (Sigma-Aldrich, USA) and stained with Syto9 and PI (Molecular Probes, USA). Ten μg mL^−1^ is a concentration equivalent to 10x MIC in this particular *P. aeruginosa*, PAO1, strain [36]. After 1, 3, 5, and 17 ​h of treatment, the wells were imaged using CLSM, as described above. The biomass of the Syto9-and PI-stained fraction were quantified based on CLSM images with pixel quantitative approach. The fraction of Syto9-stained biomass of the total biomass (X ​= ​Syto9/(PI ​+ ​Syto9)). Colony-forming unit counts were performed in wells after 24, 48, and 72 ​h of treatment. The removed biomass was washed with saline and plated as described above.

### Statistics

All statistics were calculated in Graph Pad Prism 7.0. For biomass quantification by CLSM and CFU, all data was derived from three or more biological replicates, based on three technical replicates (wells) for CLSM and two for CFU. For CV, all measurements were averaged on the basis of eight measurements. Data was tested for normality with Shapiro-Wilk Normality test. All parametric data was tested by means of a One-way ANOVA with Sidak’s multiple comparison. Kruskal-Wallis test with Dunn’s multi compassion were used for non-parametric data. P-values < 0.05 were considered significant.

## Conflicts of interests

All authors declare no conflict of interests.
